# Prognostic value of T1 substaging on oncological outcomes in patients with non-muscle-invasive bladder urothelial carcinoma: a systematic literature review and meta-analysis

**DOI:** 10.1007/s00345-019-02936-y

**Published:** 2019-09-06

**Authors:** Mehdi Kardoust Parizi, Dmitry Enikeev, Petr V. Glybochko, Veronika Seebacher, Florian Janisch, Harun Fajkovic, Piotr L. Chłosta, Shahrokh F. Shariat

**Affiliations:** 1 Department of Urology and Comprehensive Cancer Center, Vienna General Hospital, Medical University of Vienna, Währinger Gürtel 18-20, 1090 Vienna, Austria; 2 Department of Urology, Shariati Hospital, Tehran University of Medical Sciences, Tehran, Iran; 3 Institute for Urology and Reproductive Health, Sechenov University, Moscow, Russia; 4 Department for Gynecology and Gynecologic Oncology, Gynecologic Cancer Unit, Comprehensive Cancer Centre, Medical University of Vienna, Vienna, Austria; 5 Department of Urology, University Medical Center Hamburg-Eppendorf, Hamburg, Germany; 6 Department of Urology, Jagiellonian University, Medical College, Cracow, Poland; 7 Department of Urology, Weill Cornell Medical College, New York, NY USA; 8 Department of Urology, University of Texas Southwestern Medical Center, Dallas, TX USA

**Keywords:** Staging, Substage, pT1, T1, Bladder cancer, Progression, Prognosis, Urothelial carcinoma

## Abstract

**Purpose:**

To evaluate the prognostic value of substaging on oncological outcomes in patients with T (or pT1) urothelial carcinoma of the bladder.

**Methods:**

A literature search using PubMed, Scopus, Web of Science, and Cochrane Library was conducted on March 2019 to identify relevant studies according to the Preferred Reporting Items for Systematic Review and Meta-analysis (PRISMA) guidelines. The pooled disease recurrence (DR) and disease progression (DP) rate in T1(or pT1) patients were calculated using a fixed or random effects model.

**Results:**

Overall 36 studies published between 1994 and 2018 including a total of 6781 bladder cancer patients with T1(or pT1) stage were selected for the systematic review and meta-analysis. Twenty-nine studies reported significant association between tumor infiltration depth or muscularis mucosa (MM) invasion and oncological outcomes. Totally 12 studies were included in the meta-analysis. MM invasion (T1a/b/c [or pT1a/b/c] or T1a/b [or pT1a/b] substaging system) was associated with DR (pooled HR: 1.23, 95%CI: 1.01–1.49) and DP (pooled HR: 2.61, 95%CI: 1.61–4.23). Tumor infiltration depth (T1 m/e [or pT1 m/e] substaging system) was also associated with DR (pooled HR: 1.49, 95%CI: 1.11–2.00) and DP (pooled HR: 3.29, 95%CI: 2.39–4.51).

**Conclusions:**

T1(or pT1) substaging in patients with bladder cancer is of prognostic value as it is associated with oncologic outcomes. Inclusion of this factors into the clinical decision-making process of this heterogeneous tumor may improve outcomes, while avoiding over- and under-treatment for T1(or pT1) bladder cancer.

## Introduction

T1 carcinoma of the urinary bladder is a heterogeneous disease with potentially aggressive behavior leading to lethality [1]. Indeed, despite sharing many of the genetic and epigenetic factors of muscle-invasive bladder cancer, it is classified as non-muscle invasive. Yet, patients with T1 bladder cancer have an overall mortality of 33% and a cancer-specific mortality of 14% at three years after diagnosis, suggesting that these patients have a high risk of disease progression and, accordingly, require meticulous surgery, endoscopic surveillance and informed clinical decision-making [2].

The variability in the outcomes of patients with T1 bladder cancer is a result of both tumor heterogeneity and pathological staging, as well as inconsistencies in risk stratification, endoscopic resection and schedules of delivery of BCG [3]. Owing to limitations in clinical staging, patients with T1 bladder cancer are at risk of both under-treatment with use of BCG despite recurrence, and overtreatment with early radical cystectomy. Understanding the pathologic features of T1 bladder cancers and how they impact prognosis and, therefore, could improve risk stratification to align therapy with biological risk and clinical behavior of the individual tumor [4, 5]. While novel prognostic features such as variant histology and lymphovascular invasion have been included in the clinical decision-making, more features are needed to improve our prognostic accuracy [5–7].

There is a growing evidence that tumor depth and extension could be such a feature for patients with T1(or pT1) bladder cancer [8, 9]. To test this hypothesis, we performed a systematic review and meta-analysis to evaluate the value of T1(or pT1) substaging for predicting oncological outcomes in patients with T1(or pT1) urothelial carcinoma of the bladder. T1 and pT1 were referred to disease stage in patients who underwent trans-urethral resection of bladder tumor (TURBT) and radical cystectomy, respectively.

## Materials and methods

### Search strategy

A full electronic literature search using PubMed, Scopus, Web of Science, and Cochrane Library was conducted by two independent authors on March 2019 to find relevant studies for this systematic review and meta-analysis according to the Preferred Reporting Items for Systematic Review and Meta-analysis (PRISMA) guidelines [10]. The search terms used were (“T1” OR “T1a” OR “T1b” OR “T1 m” OR “T1e” OR “muscularis mucosa invasion” OR “subclassification” OR “substage” OR “substaging”) AND (“bladder cancer” OR “bladder carcinoma” OR “bladder neoplasm”). The protocol for this systematic review was registered in PROSPERO (Prospective Register of Systematic Reviews, CRD42019129661) and is available in full on the University of York website.

### Inclusion criteria

The following criteria were considered to select eligible studies: prospective or retrospective studies including full text regarding T1(or pT1) substaging in patients with non-muscle-invasive bladder cancer (NMIBC) with oncological outcomes including disease recurrence (DR) and disease progression (DP). We excluded studies in other than English, meeting abstract, case reports, review articles, replies, expert opinions, and comment letters.

### Data extraction

Data were extracted on first author, year of publication, patients, region of study, recruitment period, study design, total number of T1(or pT1) patients, number of substaged T1(or pT1) patients, substaging system, patients’ age, and follow-up duration. Oncological outcomes including DR and DP were the primary outcomes of interest. DR was defined as histological detection of bladder cancer and DP was defined as development of muscle-invasive disease or distant metastasis after primary treatment. Two independent reviewers assessed all full text studies and excluded inappropriate ones after screening based on the study title and abstract. The muscularis mucosa (MM) invasion substaging was defined as T1a/b (or pT1a/b) or T1a/b/c (or pT1a/b/c). According to the T1a/b (or pT1a/b) staging, T1a (or pT1a), where tumors cells invade the lamina propria but are still located above the level of the MM and T1b (or pT1b), where tumors cells are seen invading into or beyond the MM. In T1a/b/c (or pT1a/b/c) staging system, T1a (or pT1a) was defined as invasion into the stroma but not to MM, T1b (or pT1b); invasion into MM but not beyond MM, and pT1c (or pT1c); invasion beyond the MM but not to muscularis propria. Infiltration depth substaging system was defined as T1 m/e (or pT1 m/e). T1 m, or pT1 m (micro infiltration) was a single focus of lamina propria invasion with a maximum depth of 0.5 mm (within one high power field; objective × 40). T1e or pT1/e (extensive infiltration) was defined as a larger area with invasion or multiple micro-invasive areas.

### Statistical analyses

We extracted reported HRs and 95%CIs to calculate cumulative effect size of studies which presented the association between T1(or pT1) substaging and DR and DP. Studies presented HR using multivariate Cox proportional hazard regression model were included in meta-analysis. STATA/MPTM, version 14.2 (Stata-Corp., College Station, TX, USA) was used to perform meta-analysis. Heterogeneity between the studies included in the meta-analysis was assessed by Cochrane *Q* test and I^2^ statistics. An I^2^ > 50% and *p* value < 0.05 in Cochrane *Q* test implied that the heterogeneity existed. With no heterogeneity among selected studies, we considered fixed effect models to calculate pooled HRs. In case of significant heterogeneity, we used random effect model. Visual inspection of funnel plot was carried out to identify publication bias in our meta-analysis.

### Risk of bias (RoB) assessment

The RoB assessment of each study was done according to the Cochrane Handbook for Systematic Reviews of Interventions for including nonrandomized studies [11, 12]. The confounding factors including treatment modality, tumor grade, carcinoma in situ (CIS), multifocality, T1 (or pT1) substaging, and tumor size were identified as the most important prognostic factors. The presence of confounders was determined by consensus. The RoB assessment for each study was performed by two independent authors and the overall RoB level was presented as “low”, “intermediate”, or “high” risk.

## Results

### Literature search process

**Fig. 1:**
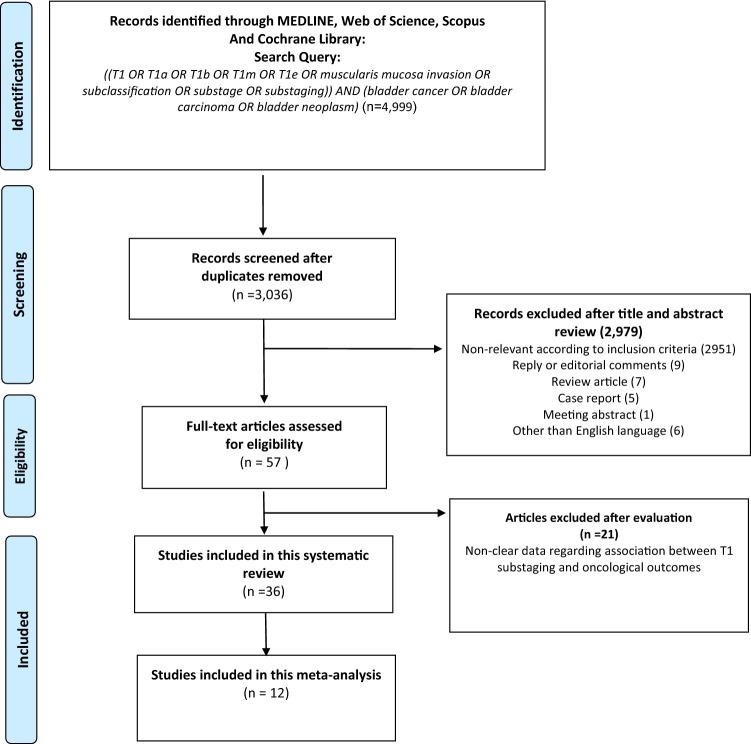
PRISMA flow chart for article selection process to analyze the prognostic value of T1 substaging on oncological outcomes in patients with non-muscle-invasive bladder urothelial carcinoma

A total of 4999 studies were found after an initial search; 3036 records remained after exclusion of duplicates (Fig. 1). After exclusion of non-relevant studies, review articles, case reports, comments, replies, meeting abstracts, and studies in other than English, 57 studies remained. Finally, 36 and 12 studies were included for qualitative and quantitative evidence synthesis, respectively.

### Characteristics of the included studies

Tables 1 and 2 summarize the studies’ characteristics and patients’ clinical data, respectively. Four studies were designed prospectively [13–16] and 32 studies were retrospective in design [8, 9, 17–46]. All studies were published between 1994 and 2018. In total, 6781 patients were included in 36 studies with 5964 patients who underwent T1 (or pT1) substaging and outcomes analysis. Twenty-three studies came from Europe, five from North America, six from Asia, and two from Europe/Canada region.

**Table 1:** Study characteristics of 40 studies assessing the prognostic value of T1 substaging in patients with bladder urothelial carcinoma

Author	Year	Region	Recruitment period	Design	No.pT1 Pts	Substaged T1 Pts	Substaging system	Oncological end point
Hasui [23]	1994	Japan	1980–1991	Retrospective	88	88	MM invasion (T1a/T1b)	DR, DP
Holmäng [24]	1997	Sweden	1987–1988	Retrospective	121	113	MM invasion (T1a/T1b)	DP, CSS, OS
Smits [40]	1998	The Netherlands	1987–1990	Retrospective	133	124	MM invasion (T1a/T1b/T1c)	DR, DP
Cheng [22]	1999	USA	1987–1992	Retrospective	83	83	Depth of lamina propria invasion	DP
Kondylis [26]	2000	USA	1981–1997	Retrospective	55	49	MM invasion (T1a/T1b)	DR, DP
Shariat [39]	2000	USA	N/A	Retrospective	47	36	MM invasion (T1a/T1b)	DR, DP, OS
Bernardini [17]	2001	France	1973–1996	Retrospective	149	94	MM invasion (T1a/T1b)	PFS
Sozen [42]	2002	Turkey	1983–1997	Retrospective	90	50	MM invasion (T1a/T1b)	DR, DP
Orsola [32]	2005	Spain	1996–2001	Retrospective	97	85	MM invasion (T1a/T1b/T1c)	RFS, PFS
van der Aa [45]	2005	The Netherlands	N/A	Retrospective	63	53	Tumor infiltration depth (T1 m/T1e)	DP
Chaimuangraj [20]	2006	Thailand	1990–2004	Retrospective	192	192	Muscularis mucosa invasion	DR
Andius [13]	2007	Sweden	1987–1988	Prospective	121	121	MM invasion (T1a/T1b)^†^	PFS, CSS
Mhawech-Fauceglia [29]	2007	Switzerland	N/A	Retrospective	45	45	MM invasion (T1a/T1b)	DR, DP
Queipo-Zaragoza [37]	2007	Spain	1986–2003	Retrospective	91	83	MM invasion (T1a/T1b)	DP
Soukup [16]	2008	Czech Republic	2001–2005	Prospective	105	99	MM invasion (T1a/T1b)	DR, DP (PFS)
Orsola [14]	2010	Spain	N/A	Prospective	159	138	MM invasion (T1a/T1b)	DR, DP
Bertz [18]	2011	Germany	1989–2006	Retrospective	309	309	MM invasion (T1a/T1b), Infiltration depth (≤ 1 HPF/> 1 HPF)	CSS, RFS, PFS
Palou [34]	2012	Spain/Belgium	1985–1996	Retrospective	146	93	MM invasion (T1a/T1b/T1c)	DR, DP, CSM
Lee [27]	2012	Korea	1999–2009	Retrospective	183	183	MM invasion (T1a/T1b/T1c)	DR, DP, CSM
Chang [21]	2012	Taiwan	1991–2005	Retrospective	509	509	Muscularis mucosa invasion, Infiltration depth (3 cut-off values to substage the T1 tumors: 0.5 mm, 1.0 mm, and 1.5 mm)	DR, DP, CSD, OM
van Rhijn [46]	2012	The Netherlands/Canada	1984–2006	Retrospective	129	129	MM invasion (T1a/T1b/T1c), tumor infiltration depth (T1 m/T1e)	DR, DP
Brimo [19]	2013	Canada	2004–2012	Retrospective	86	86	Muscularis mucosa invasion, Maximum tumor depth (mm)	DR,DP,WFS
Olsson [31]	2013	Sweden	1992–2001	Retrospective	285	211	MM invasion (T1a/T1b/T1c)	DR, DP
Nishiyama [30]	2013	Japan	1995–2010	Retrospective	79	79	Tumor infiltration depth (T1 m/T1e)	DR, DP
Rouprêt [38]	2013	France	1994–2010	Retrospective	612	587	MM invasion (T1a/T1b)	RFS, PFS, CSS
Soukup [41]	2014	Czech Republic	2002–2009	Retrospective	200	176	MM invasion (T1a/T1b)	RFS, PFS, CSS, OS
Hu [25]	2014	USA	1997–2005	Retrospective	39	23	Focality, Percentage of tumor invasion, and aggregate length of invasion	DR
D. E. Marco [44]	2014	Italy	2000–2006	Retrospective	40	40	MM invasion (T1a/T1b/T1c), tumor infiltration depth (T1 m/T1e)	CSS, DP
Lim [28]	2015	Korea	1998–2012	Retrospective	177	141	MM invasion (T1a/T1b/T1c)	RFS, PFS
Orsola [15]	2015	Spain	N/A	Prospective	200	200	MM invasion (T1a/T1b)	DR, DP
Patschan [36]	2015	Sweden	1997–2003	Retrospective	167	152	MM invasion (T1a/T1b/T1c)	PFS
Patriarca [35]	2016	Italy	2011–2007	Retrospective	450	314	MM invasion (T1a/T1b), tumor infiltration depth (T1 m/T1e), ROL substaging^†^	DR, DP
Colombo [8]	2018	Italy	2007–2011	Retrospective	502	250	MM invasion (T1a/T1b/T1c), microinfiltration and extended infiltration of LP (T1 m/T1e), ROL substaging	DR, DP
Fransen van de Putte [9]	2018	Europe/Canada	1982–2010	Retrospective	601	601	MM invasion (T1a/T1b), microinfiltration and extended infiltration of LP (T1 m/T1e)	PFS, CSS
Otto [33]	2018	Germany/The Netherlands	1989–2012	Retrospective	322	322	Metric T1 substage (tumor infiltration depth)	PFS, CSS, OS
Turan [43]	2018	Turkey	2009–2014	Retrospective	106	106	MM invasion (T1a/T1b), tumor infiltration depth (T1 m/T1e)	DR, DP

*N/A* not available, *LP* lamina propria, *MM* muscularis mucosa, *PFS* progression-free survival, *CSM* cancer-specific mortality, *CSS* cancer-specific survival, *OS* overall survival, *WFS* worsening-free survival, *DR* disease recurrence, *DP* disease progression, *RFS* recurrence-free survival, *OM* overall mortality, *HPF* high power field

^†^ROL substaging ROL1 < 1 power field (objective 20×, ocular 10×/field 22, diameter 1.1 mm) of invasion, approximately corresponding to invasion of the lamina propria 1 mm thick or less; ROL2: > 1 power field (objective 20×), approximately corresponding to invasion of the lamina propria more than 1 mm thick, or multifocal invasion with foci cumulatively amounting to invasion of the lamina propria more than 1 mm thick

**Table 2:** Patient characteristics in 40 studies assessing the prognostic role of T1 substaging in patients with bladder urothelial carcinoma

Author	Age, year (range)	Independent correlation with oncologic outcomes	Follow-up duration
Hasui [23]	Mean: 68 (37–95)	S	N/A
Holmäng [24]	Mean: 73.1 (48–97)	S (for DP and CSS)	≥ 5 years
Smits [40]	N/A	S (for PFS)	Minimal follow-up: 3 years
Cheng [22]	Mean: 71 (47–94)	S	Mean: 5.2 years (range, 1 day–10.4)
Kondylis [26]	N/A	NS	Median 71 months (range, 4–147)
Shariat [39]	Median: 67 (30–86)	NS	Median: 79 months
Bernardini [17]	Mean: 68.9 (42–90)	S	Mean: 64.9 months (range, 5–288)
Sozen [42]	Median: 62 (33–84)	S	Mean: 68 months (range, 24–120)
Orsola [32]	Mean: 66.4(30.3–86.8)	S (in T1b/c vs T1a substaging for RFS and PFS)	Mean: 53 months
van der Aa [45]	Mean: 68 (47–90)	S	Median: 55 months (range, 9–228)
Chaimuangraj [20]	Mean: 60 (43–83)	S	N/A
Andius [13]	Median: 74 (48–98)	NS	Median: 15 years for alive cases
Mhawech-Fauceglia [29]	Mean: 70	S (for DP)	Median: 12 months
Queipo-Zaragoza [37]	Mean: 68.1	S	Mean: 57.8 months (range, 13–24)
Soukup [16]	Mean: 68.43 (38–87)	S (for PFS)	Mean: 23.31 months
Orsola [14]	Mean: 69	S (for DP)	Median: 20.3 months
Bertz [18]	Median: 71.7 (38–87 years).	S (in Infiltration depth: ≤ 1 HPF vs > 1 HPF for RFS and PFS)	Mean: 49 months (range, 5–172)
Palou [34]	Mean: 64.9 (25–81)	NS	Median: 8.7 years
Lee [27]	Mean: 63.5 years (27–93)	S (for DP and CSM)	Mean: 43.5 months (range, 12–146)
Chang [21]	Mean: 71 (23–92)	S (MM invasion: S for DP, CSM, and OM) (depth of high-grade tumor: S for DR, DP, CSM, OM)	Mean: 88 months (range, 1–240) for patients who were alive Mean: 39 months (range, 1–193) for patients who died
van Rhijn [46]	Mean: 68.8	S (in T1 m/T1e for DP)	Median: 6.5 years
Brimo [19]	Mean: 71	S	Mean: 29 months
Olsson [31]	Median: 74	S (in T1b/c vs T1a substaging for DP in patients older than 73 years)	Median: 60 months
Nishiyama [30]	Mean: 68.5	S (for DR)	Mean: 74.0 months
Rouprêt [38]	Median: 70	S	Mean: 44 months (range, 6–161)
Soukup [41]	Median: 68.83 (17.55–86.94)	S (for PFS, CSS, OS)	Median: 3.13 years (0.1–10.5)
Hu [25]	Mean: 70 years (56–94)	S (in aggregate length of invasion; > 0.5 cm)	N/A
D. E. Marco [44]	Mean: 69.9	NS	Median: 9.5 years
Lim [28]	Mean: 68.9 (20–93)	S (for PFS)	Mean: 73.3 months (range, 3.9–187.9)
Orsola [15]	Median: 71	S (for DP)	Median: 71 months (range: 5–107)
Patschan [36]	Median: 74	NS	(3 years follow-up in analysis)
Patriarca [35]	Mean: 71.3 (64–79)	S (in ROL1 VS ROL 2 substaging for DP)	Mean: 46 months
Colombo [8]	Mean: 70 (64–77)	S (for DP in ROL2 vs ROL1 substaging)	Median: of 60 months
Fransen van de Putte [9]	Median: 71	S (for PFS and CSS in T1e vs T1 m substaging)	Median: 5.9 years
Otto [33]	Median: 72	NS	Median: 42 months
Turan [43]	Mean: 67.9	S (in T1a/b substaging for DR)	Mean: 54 months

*N/A* not available, *S* significant, *NS* non-significant, *MM* muscularis mucosa *PFS* progression-free survival, *CSM* cancer-specific mortality, *CSS* cancer-specific survival, *OS* overall survival, *OM* overall mortality, *DR* disease recurrence, *DP* disease progression, *RFS* recurrence-free survival, *HPF* high power field

^†^*S* statistical significance *p* value < 0.05

Nine studies included patients who had been substaged with both MM and tumor infiltration depth staging systems. Twenty-two studies included MM invasion substaging system only and five included patients substaged with tumor infiltration depth staging system only. TURBT with or without intravesical BCG or chemotherapy agents was reported as initial therapy in 6677 patients. Radical or partial cystectomy and/or radiation therapy were reported in 104 patients as initial therapeutic modality [13, 17, 24, 29, 35, 39, 45]. The prognostic value of T1(or pT1) substaging on at least one oncological outcome was established in 29 studies.

### Meta-analysis

#### T1 (or pT1) MM invasion substaging and DP

**Fig. 2:**
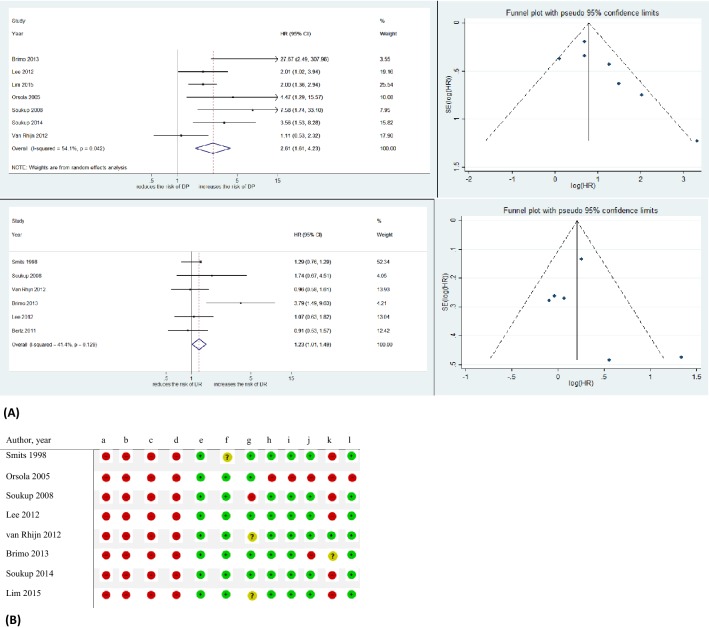
**A** Forest plots and funnel plot of studies investigating the association of T1a/b/c substaging system with disease progression (DP) and disease recurrence (DR) outcomes. **B** T1a/b/c substaging system RoB table, **a** Random sequence generation (selection bias). **b** Allocation concealment (selection bias). **c** Blinding of participants and personnel (Performance bias.). **d** Blinding of outcome assessment (detection bias). **e** Incomplete outcome data (attrition bias). **f** Selective reporting (reporting bias); and adjustment for the effects of the following confounders. **g** Treatment modality. **h** Tumor grade. **i** CIS. **j** Multifocality. **k** T1 m/e substaging. **l** Tumor size. Green circles: low risk of bias and confounding, red circles: high risk of bias and confounding, yellow circles: unclear risk of bias and confounding. *CI* confidence interval, *HR* hazard ratio

The impact of MM invasion on DP was investigated in patients with T1(or pT1) bladder urothelial carcinoma. Overall seven studies with a total of 899 patients were identified and MM invasion was associated with a higher DP rate (pooled HR 2.61, 95%CI: 1.61–4.23) (Fig. 2A) [16, 19, 27, 28, 32, 41, 46]. A statistically significant heterogeneity was found among included studies using the Chi-square and *I*^2^ tests (I^2 = 54.1%, *p* = 0.042); the weights were from random effect model to analyze pooled HR. Funnel plots identified one study over the pseudo 95%CI (Fig. 2A).

#### T1 (or pT1) MM invasion substaging and DR

Six studies in a total of 930 patients reported HR to present the prognostic value of MM invasion on DR in T1(or pT1) urothelial bladder carcinoma patients [16, 18, 19, 27, 40, 46]. The overall pooled HR was 1.23 (95%CI: 1.01–1.49) implying a significant association between MM invasion and DR (Fig. 2A). The Chi-square and *I*^2^ tests did not show any significant heterogeneity (I^2 = 41.4%, *p* = 0.129). Funnel plots revealed one study over the pseudo 95%CI (Fig. 2A). Figure 2B shows the RoB table of studies included in the T1(or pT1) MM invasion substaging meta-analysis.

#### Infiltration depth substaging and DP

**Fig. 3:**
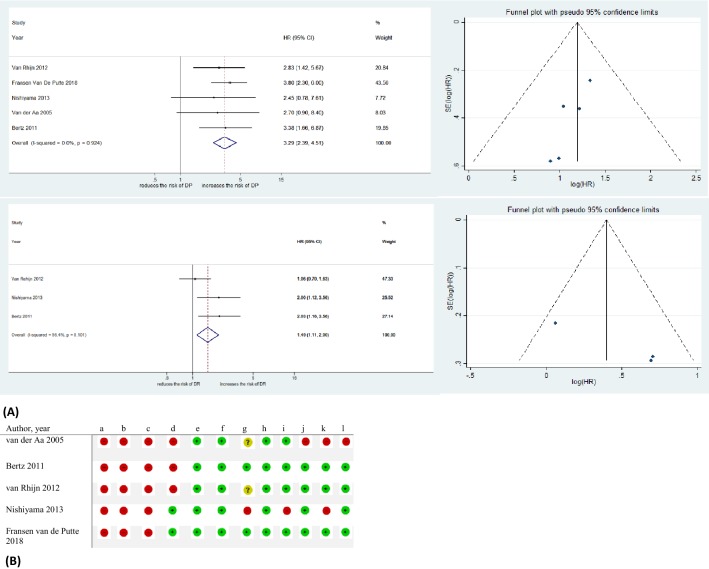
**A** Forest plots and funnel plot of studies investigating the association of T1 m/e substaging system with disease progression (DP) and disease recurrence (DR) outcomes. **B** T1 m/e substaging system RoB table, **a** Random sequence generation (selection bias). **b** Allocation concealment (selection bias). **c** Blinding of participants and personnel (Performance bias.). **d** Blinding of outcome assessment (detection bias). **e** Incomplete outcome data (attrition bias). **f** Selective reporting (reporting bias); and adjustment for the effects of the following confounders. **g** treatment modality. **h** tumor grade. **i** CIS. **j** Multifocality. **k** T1a/b/c substaging. **l** Tumor size. Green circles: low risk of bias and confounding, red circles: high risk of bias and confounding, yellow circles: unclear risk of bias and confounding. *CI* confidence interval, *HR* hazard ratio

Five studies with a total of 1171 patients with T1(or pT1) bladder urothelial carcinoma reported the association of tumor infiltration depth and DP [9, 18, 30, 45, 46]. Tumor infiltration depth was associated with DP (pooled HR: 3.29, 95%CI: 2.39–4.51) (Fig. 3A). There was no significant heterogeneity in the Cochrane *Q* or *I*^2^ tests (I^2 = 0.0%, *p* = 0.924). No study was detected over the pseudo 95%CI on Funnel plots (Fig. 3A).

#### Infiltration depth substaging and DR

The impact of infiltration depth on DR was investigated in three studies in a total of 517 patients with T1(or pT1) bladder urothelial carcinoma [18, 30, 46]. There was a significant association between infiltration depth and DR with pooled HR of 1.49 (95%CI: 1.11–2.00) (Fig. 3A). The Chi-square and *I*^2^ tests did not show any significant heterogeneity (I^2 = 56.4%, *p* = 0.101). Funnel plots identified no study over the pseudo 95%CI (Fig. 3A). Figure 3B shows the RoB table of studies included in T1(or pT1) Infiltration depth substaging meta-analysis.

## Discussion

In this systematic review and meta-analysis, we assessed the prognostic value of T1(or pT1) substaging systems on oncological outcomes in patients with T1(or pT1) bladder urothelial carcinoma. Both MM invasion and tumor infiltration depth substaging systems were strongly associated with both DR and DP after adjusting for the effects of established confounding factors (e.g., tumor grade, CIS, and multifocality).

The most widely used prognostic tools, taking into account tumor grade and stage, prior recurrences, tumor size, multifocality, and the presence of CIS, are still suboptimal to predict DR and DP. Moreover, the lack of effective bladder cancer information among general public may be as an important factor affecting patients’ outcomes and online information and social media could be effective to improve quality of patient’s care and disease management in patients with bladder cancer [47].

We and others have shown that the current prognostic and risk stratification tools are too inaccurate to guide clinical decision making safely [1, 48, 49]. In this review and meta-analysis, we confirm that tumor invasion into MM and tumor infiltration depth of more than 0.5 mm are strong predictors of disease recurrence and progression and could be used to distinguish high risk patients for recurrence and progression who might benefit from standard adjuvant therapy (e.g., intravesical immunotherapy or chemotherapy). From these who are most likely to benefit from intensification of care such as early radical cystectomy.

In patients with NMIBC, the probability of disease progression can be as high as 45% at five years [50]. Although it has been suggested that MM substaging might be helpful to identify high risk patients who are likely to suffer from disease progression despite adequate intravesical therapy, available data quality has not been of high quality and prognostic tools have not included this valuable parameter [38, 51]. Martin-Doyle et al. evaluated the prognosticators to improve selection criteria for early cystectomy in patients with high-grade T1 bladder cancer in a meta-analysis. The authors reported T1a/b substaging system as a valuable prognosticator of oncological outcomes comparable with our study with pooled HR of 1.81 (95%CI: 0.88–3.73) for DR and pooled HR of 3.55 (95%CI: 1.92–6.56) for DP in 420 and 785 patients with high-grade T1 bladder cancer, respectively [51]. We confirmed that both MM invasion and tumor infiltration depth are strong predictors of disease progression after controlling for the effect of standard prognosticators. Indeed, patients harboring T1b/c or T1e in substaging system may benefit from early radical cystectomy as their tumor carries the biologic and clinical behavior of muscle-invasive bladder cancer [51]. In patients considered candidates for radical cystectomy, pretreatment imaging modalities including magnetic resonance imaging and positron emission topography/computed tomography (CT) provide higher sensitivity and similar specificity compared to CT for detection of positive lymph nodes that might have a significant impact on clinical decision-making process [52].

A consensus among pathologists is urgent to propose T1(or pT1) substaging systems as a prognosticator in TNM classification system and guidelines. MM is identified in 12–83% of bladder biopsy specimen [53, 54]. Therefore, some studies proposed identification of large vessels of the vascular plexus as an alternative tumor extension marker in specimens without obvious MM [43, 46]. Moreover, although a cut-off point of 5 mm has been proposed in several studies to define tumor infiltration depth, other studies have utilized other definitions [8, 35]. These discrepancies between definitions may lead to low reproducibility and questionable validity. Standardization and prospective assessment in controlled studies is necessary.

According to our study, although substaging of T1(or pT1) disease is somewhat controversial and difficult to implement in all cases; the main advantage of this scoring system is to identify the high risk T1 bladder cancer patients who might benefit from more rigorous follow-up and ideally from more aggressive treatments which are appropriate for invasive bladder carcinoma.

This study is not without limitations. The majority of included studies in this systemic review were retrospective in design precluding robust conclusions about the prognostic value of T1(or pT1) substaging systems. Moreover, the heterogeneity of substaging systems was found in MM invasion and tumor infiltration depth systems as well as the outcomes assessed in the studies makes clear conclusions difficult. Indeed, further studies are needed to assess the prognostic value of T1(or pT1) substaging systems in patient counselling and risk-based selection of the personalized therapeutic modality.

## Conclusion

We found that T1(or pT1) substaging systems are strong predictors of oncological outcomes (DR, DR). Although T1(or pT1) substaging systems are promising and can be used as an aid in determining the most appropriate treatment modality and intensity of follow-up, optimal T1(or pT1) substaging system definition remains to be elucidated in future well-designed prospective studies.
